# Acute Respiratory Distress Syndrome Associated With *Listeria monocytogenes* in a Pregnant Woman: Case Report and Systematic Review

**DOI:** 10.1155/crcc/9923135

**Published:** 2025-10-29

**Authors:** Cristian Morán Mariños, Renzo Villanueva-Villegas, Carlos Quispe-Vicuña, Gabriela Bedoya Tapia, Kimberly López-Pilco

**Affiliations:** ^1^ Department of Pulmonology, Hospital Nacional Dos de Mayo, Lima, Peru; ^2^ Research Unit in Bibliometrics, Vice-Rectorate for Research, Universidad San Ignacio de Loyola, Lima, Peru, usil.edu.pe; ^3^ Research Group in Neurosciences, Metabolism, Clinical and Health Effectiveness (NEMECS), Universidad Científica del Sur, Lima, Peru, cientifica.edu.pe; ^4^ Intensive Care Unit, Hospital Nacional Dos de Mayo, Lima, Peru

**Keywords:** case reports, *Listeria monocytogenes*, pregnancy, respiratory distress syndrome, systematic review

## Abstract

**Purpose:**

The purpose of the study is to describe the clinical presentation of pregnant women diagnosed with acute respiratory distress syndrome (ARDS) secondary to *Listeria monocytogenes* infection, complemented by a systematic review of reported cases in the literature.

**Materials and Methods:**

A systematic review was conducted in accordance with PRISMA guidelines using major international databases (PubMed, Scopus, Web of Science, Embase, and SciELO) and gray literature sources. Included studies comprised case reports of ARDS associated with microbiologically confirmed gestational listeriosis. Data extraction focused on clinical, laboratory, and imaging variables, as well as maternal–fetal outcomes.

**Results:**

A total of three prior cases of ARDS associated with gestational listeriosis were identified in the literature. Including the present report, four cases were analyzed, with a mean maternal age of 26 years. Three patients were in the third trimester and one in the second trimester. The duration of illness ranged from 3 to 6 days, with all patients presenting with fever and tachypnea; additional symptoms included abdominal pain, nausea, vomiting, chest pain, and dyspnea. Laboratory abnormalities included lymphopenia (244–393 cells/mm^3^) and thrombocytopenia (56,000–68,000/mm^3^) in two patients. Three required intensive care monitoring, and two underwent mechanical ventilation. Maternal outcomes were favorable in all cases; however, one fetal death was reported.

**Conclusion:**

ARDS associated with listeriosis during pregnancy is an exceptionally rare but high‐risk condition that necessitates early diagnosis and timely intensive care. This study highlights the importance of prompt clinical recognition, appropriate antibiotic therapy with ampicillin, and the potential role of noninvasive oxygenation strategies to avoid intubation in pregnant patients with acute respiratory failure.

## 1. Introduction

Listeriosis is an infectious disease caused by *Listeria monocytogenes*, an intracellular pathogen primarily transmitted through contaminated food. This infection poses a particular risk during pregnancy, as pregnant women are up to 18 times more likely to contract the disease compared to the general population, especially during the third trimester [1]. Globally, the reported incidence of listeriosis ranges from 5 to 13.7 cases per 100,000 deliveries, with an estimated overall mortality rate of 13% [2–4]. Despite the elevated risk, fewer than 2.3% of pregnant individuals develop respiratory symptoms or ARDS as a complication of listeriosis [5].

In addition to this rare manifestation, pregnant patients may also experience sepsis and septic shock and require admission to intensive care units (ICUs) [5]. Fetal consequences of maternal listeriosis can be severe, including fetal distress, preterm delivery, or even fetal death [6]. Therefore, timely diagnosis and appropriate treatment are essential to minimize risks to both mother and fetus [7].

Due to the rarity of this complication and the limited number of reported studies, the objective of the present study was to conduct a systematic review of ARDS cases in pregnant women with listeriosis, complemented by the report of a Peruvian case. Through this analysis, we aim to provide an integrated perspective on the clinical and epidemiological characteristics of the condition, contributing to a better understanding and management of this severe but infrequent complication in the context of gestational listeriosis.

## 2. Case Report

A 27‐year‐old Venezuelan woman, at 17 weeks of gestation confirmed by first‐trimester ultrasound, presented for medical evaluation after developing symptoms 2 days following the consumption of food from a local market in Lima, Peru. She reported a 3‐day history of fever, general malaise, colicky abdominal pain, nausea, and vomiting. During a prior consultation, she had been diagnosed with a urinary tract infection and prescribed cefuroxime and oral hydration. However, due to persistent symptoms, she sought care at the gynecology and obstetrics emergency department.

Upon admission, her vital signs were as follows: blood pressure 130/85 mmHg, heart rate 108 bpm, respiratory rate 24 breaths per minute, and temperature 38.3°C. Physical examination revealed preserved vesicular breath sounds with no added noises, rhythmic heart sounds without murmurs, and audible fetal heartbeats on obstetric assessment. She was admitted to the hematology service with a presumptive diagnosis of pancytopenia, under evaluation for possible autoimmune disease or urinary sepsis, based on laboratory findings of anemia (Hb: 11.9 g/dL), leukopenia (3250 cells/mm^3^), lymphopenia (393 cells/mm^3^), and thrombocytopenia (68,000 platelets/mm^3^).

During the first day of hospitalization, her condition worsened with progression of dyspnea from MMRC Grade 1 to Grade 3, respiratory rate increased to 32 breaths per minute, and she required supplemental oxygen via Venturi mask (FiO_2_ at 40%). Arterial blood gas analysis showed oxygen saturation of 93.5%, PaO_2_ of 67 mmHg, and PaCO_2_ of 26.8 mmHg, with a PaO_2_/FiO_2_ ratio of 134. Based on these findings, she was transferred to the ICU with a presumptive diagnosis of moderate respiratory failure, possibly due to atypical pneumonia, and high‐flow nasal cannula therapy was initiated (60 L/min, FiO_2_ at 80%).

**Figure 1:**
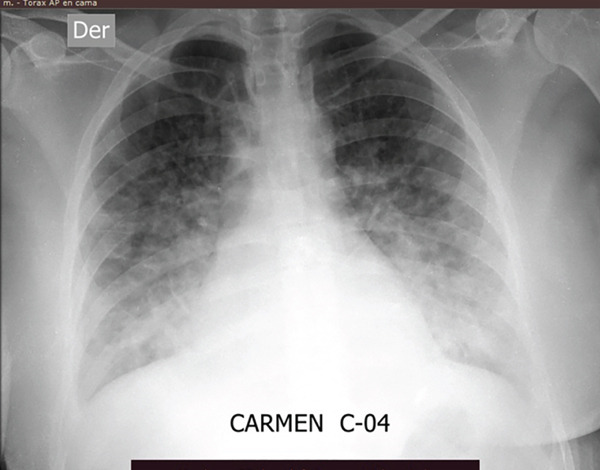
Chest x‐ray of a 17‐week pregnant woman with ARDS associated with *Listeria monocytogenes*.

Serologic tests for HIV, HBV, HCV, and syphilis were negative, as were the viral and atypical pathogen panels. On the third day of hospitalization, blood cultures returned positive for *Listeria monocytogenes*. Chest radiography revealed reticular and alveolar infiltrates in the anterior and lower segments of both lung fields, along with obliteration of both costophrenic angles (Figure 1). Antibiotic treatment was initiated with intravenous ampicillin at a dose of 2 g every 4 h.

By the fifth day, the patient showed clinical improvement in respiratory function and was transferred back to the gynecology‐obstetrics service. She completed a 14‐day course of intravenous ampicillin and was discharged without complications for both herself and the fetus. Follow‐up confirmed an uncomplicated term delivery at 37 weeks of gestation.

## 3. Materials and Methods

This systematic review was conducted in accordance with the Preferred Reporting Items for Systematic Reviews and Meta‐Analyses (PRISMA) guidelines, with a protocol registered in PROSPERO (ID: CRD420251107661) [8]. We searched PubMed, Scopus, Web of Science, Embase, and SciELO up to August 2024, complemented by gray literature through Google Scholar.

Eligible studies included case reports or case series describing pregnant women with microbiologically confirmed *Listeria monocytogenes* infection complicated by ARDS or acute respiratory failure. Two reviewers independently screened the records, and discrepancies were resolved by consensus.

Extracted data covered clinical characteristics, laboratory findings, imaging results, and maternal–fetal outcomes. Given the rarity of the condition and the limited number of available studies, findings were summarized descriptively in a comparative table.

Table S1 provides the complete search strategies for each database, structured around three core concepts (*Listeria monocytogenes*, acute respiratory distress syndrome, and pregnancy), with syntax adapted to the requirements of each platform.

## 4. Results

### 4.1. Systematic Review

**Figure 2:**
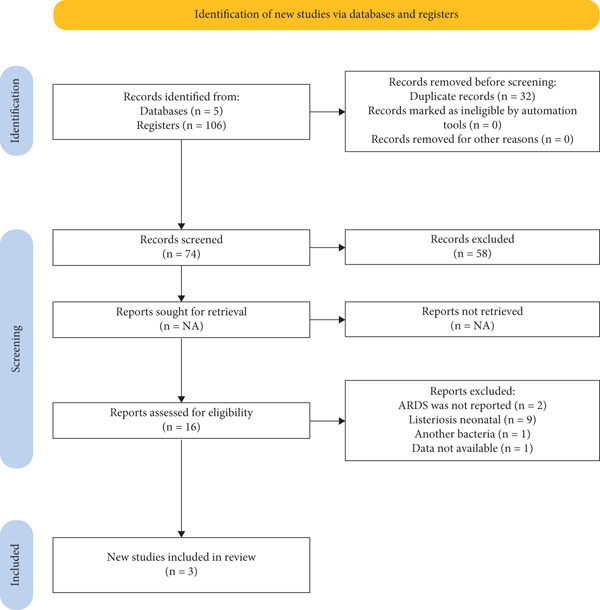
Flow diagram of selected studies.

A total of 106 records were retrieved, of which 32 duplicates were removed. After screening the titles and abstracts of the remaining 74 studies, 16 full‐text articles were assessed for eligibility, and ultimately 3 studies were included in the final analysis (Figure 2). One additional article, which reported what is believed to be the first case of ARDS in a pregnant woman with listeriosis, was excluded due to insufficient data availability [9].

### 4.2. Characteristics of Included Studies

A total of four patients were identified, including the present case, with a mean age of 26 years. Regarding gestational age, three patients were in the third trimester and one in the second trimester. The duration of illness ranged from 3 to 6 days, during which all patients experienced fever and tachypnea. Other reported symptoms included abdominal pain, nausea and vomiting, chest pain, and dyspnea. All cases fulfilled diagnostic criteria for ARDS, based on blood gas analyses and radiologic findings. Two of the four patients had lymphopenia (393–244 cells/mm^3^) and thrombocytopenia (56,000–68,000 platelets/mm^3^). Three of the women required intensive care monitoring, and two underwent endotracheal intubation. All patients were discharged with favorable clinical outcomes (Table 1).

**Table 1:** Characteristics of the reported studies.

**Author**	**Year**	**Age**	**Gestational age (weeks)**	**Duration of illness**	**Vital signs**	**Signs and symptoms**	**Laboratory findings**	**Imaging findings**	**Complications**	**Outcome**
C. Moran	2025	27	27	3 days	HR: 108 bpm RR: 32 bpm T: 38.3°C	Abdominal pain Nausea and vomiting Fever	‐Hb: 11.9 g/dL ‐WBC: 3250/mm^3^ ‐Lymphocytes: 393/mm^3^ ‐Platelets: 68,000/mm^3^ ‐PO_2_: 67 mmHg ‐PCO_2_: 26.8 mmHg ‐PaO_2_/FiO_2_: 134 ‐FiO_2_: 50%	Chest x‐ray: Bilateral reticular and alveolar infiltrates in anterior and lower lung fields, with obliteration of both costophrenic angles	‐ICU	Discharged with favorable recovery
Huang et al. [10]	2023	24	29	NR	T: 39.5°C HR: NR RR: NR	Abdominal pain Chest pain Fever	‐Hb: 8 g/dL ‐WBC: 14.23 × 10^9^/L ‐Lymphocytes: 1.05 × 10^9^/L ‐Platelets: NR ‐pH: NR ‐PO_2_: 67 mmHg ‐PCO_2_: NR ‐PaO_2_/FiO_2_: NR ‐FiO_2_: Binasal cannula (NR)	Chest CT: Enlarged cardiac silhouette, pericardial effusion, bilateral ground‐glass opacities with irregular consolidation, and thickened bronchovascular bundles	‐ICU ‐Septic shock ‐Emergency cesarean section ‐Fetal death ‐Endotracheal intubation	Discharged with favorable recovery
Sepúlveda‐Bajo et al. [11]	2005	34	19	6 days	T: 39°C HR: NR RR: NR	Dyspnea Fever	‐Hb: 8.7 g/dL ‐WBC: 9400/mm^3^ ‐Lymphocytes: NR ‐Platelets: NR ‐pH: 7.49 ‐PO_2_: 44.2 mmHg ‐PCO_2_: 27 mmHg ‐PaFi: 211 ‐FiO_2_: 21%	Chest x‐ray: Bilateral interstitial alveolar infiltrates	‐Sepsis	Discharged with favorable recovery
Boucher et al. [12]	1984	20	33	4 days	HR: > 100 bpm RR: 56 bpm T: 42°C	Shallow breathing Fever	‐Hematocrit: 27% ‐WBC: 6100/mm^3^ ‐Lymphocytes: 244/mm^3^ ‐Platelets: 56,000/mm^3^ ‐pH: 7.38 ‐PO_2_: 58 mmHg ‐PCO_2_: 33 mmHg ‐PaFi: 58 ‐FiO_2_: 100%	Chest x‐ray: Bilateral pulmonary infiltrates predominantly affecting the lower lobe of the right lung, with obliteration of the right hemidiaphragm	‐Septic shock ‐Disseminated intravascular coagulation ‐Endotracheal intubation	Discharged with favorable recovery and healthy fetus

Abbreviation: NR, not reported.

### 4.3. Discussion

Acute respiratory distress syndrome is a well‐recognized critical complication in the context of general and maternal sepsis. However, its occurrence in pregnant women with listeriosis is exceptionally rare and has been scarcely documented in the medical literature. *Listeria monocytogenes* infection poses a significant threat during pregnancy, with a high potential for maternal and fetal morbidity and mortality [13]. Although the initial clinical presentation is often nonspecific—typically involving fever, gastrointestinal symptoms, or general malaise—it can progress in severe cases to sepsis, fetal demise, or ARDS, as demonstrated in the present case.

*Listeria monocytogenes* is a facultative intracellular Gram‐positive bacillus primarily transmitted through the ingestion of contaminated food. It shows marked tropism for placental tissue and the central nervous system, which explains its ability to breach immunological barriers and compromise pregnancy [3, 14].

In our case, a 17‐week pregnant woman developed ARDS with hypoxemia (PaO_2_/FiO_2_ = 134), lymphopenia, thrombocytopenia, and bilateral infiltrates on chest radiography. Blood cultures confirmed *Listeria monocytogenes*, and she improved with high‐flow nasal cannula and intravenous ampicillin without requiring intubation.

When compared with the three previously reported cases of ARDS in pregnancy due to listeriosis [10–12], several similarities were observed: all presented with fever, acute respiratory compromise, and bilateral pulmonary infiltrates, and all achieved favorable maternal outcomes under intensive care and targeted antibiotic therapy. Differences included gestational age (two in the third trimester, one in the second, and our case also in the second), the degree of hypoxemia, and ventilatory support requirements—two required invasive mechanical ventilation, whereas ours and one Spanish case improved without intubation. Fetal outcomes also varied: while three pregnancies progressed favorably, one fetal death occurred in a twin gestation [10]. This low frequency may reflect the genuinely rare occurrence of the event or could be attributed to underreporting due to low clinical suspicion or limited use of imaging studies in pregnant women with listeriosis.

ARDS is a recognized complication of maternal sepsis, but in the context of listeriosis, it remains exceptional. In a cohort of 66 pregnant women admitted to the ICU for sepsis of various causes, Timezguid et al. reported ARDS in 17% of cases, with nearly one‐third requiring mechanical ventilation and one‐quarter needing vasopressors [15]. In contrast, Craig et al., analyzing 666 hospitalizations for gestational listeriosis, found ARDS in only 2.8% of infected patients, compared with 0.1% in uninfected controls, while the rates of mechanical ventilation and shock were markedly lower (1.4% each) [5]. These data emphasize that although *Listeria monocytogenes* can trigger severe systemic complications, its progression to ARDS is a rare manifestation, underscoring the need for high clinical suspicion in pregnant women with systemic symptoms and relevant dietary exposures.

In terms of diagnosis, chest radiography was consistently useful across our case and the three prior reports, demonstrating bilateral diffuse infiltrates typical of ARDS. This first‐line tool remains safe during pregnancy when performed with standard precautions and often obviates the need for advanced imaging. Computed tomography may provide additional detail but should be reserved for selected cases, as the diagnostic yield of plain radiography was sufficient to guide early recognition and targeted therapy in all reported patients [16–18]. Collectively, these findings reinforce the importance of prompt imaging, microbiologic confirmation, and immediate initiation of ampicillin‐based regimens in improving maternal outcomes.

From a pathophysiological perspective, *Listeria monocytogenes* can induce ARDS through hematogenous dissemination, activation of the innate immune response, and disruption of the alveolar–capillary barrier [19]. Pregnancy amplifies these mechanisms due to increased plasma volume, reduced oncotic pressure, diminished functional residual capacity, and a Th2‐biased immune shift that favors intracellular pathogens, predisposing to severe hypoxemia and alveolar collapse [20].

Maternal oxygenation and ventilation must therefore be managed with caution. Excessive ventilation leading to hypocapnia may cause uterine vasoconstriction and compromise fetal perfusion [21]. In our case, high‐flow nasal cannula was initiated, improving oxygenation and avoiding intubation. This strategy has been proposed as an effective option in mild to moderate acute respiratory failure and may be particularly advantageous in obstetric patients, although it requires close monitoring to prevent delayed recognition of treatment failure [22–24]. Overall, ARDS secondary to gestational listeriosis remains a rare but high‐risk complication. Current evidence supports early recognition and treatment with high‐dose intravenous ampicillin as the cornerstone of therapy [14, 25]. In severe cases, ventilatory support should not be delayed, but management must balance maternal oxygenation with fetal well‐being. Prompt diagnosis, targeted antibiotic therapy, and appropriate respiratory support remain essential to improve outcomes in this exceptional clinical scenario.

#### 4.3.1. Strengths and Limitations

This study combines a detailed case report with a structured literature search to synthesize the scarce evidence on ARDS secondary to gestational listeriosis. The approach enhances transparency in case identification and data extraction. Evidence is limited to four cases overall, reflecting rarity and precluding robust statistical analysis or formal risk‐of‐bias assessment. Heterogeneity and incomplete reporting across case reports further restrict direct comparisons. Despite these constraints, the synthesis offers practical signals to aid early recognition and management.

#### 4.3.2. Conclusion

ARDS associated with listeriosis during pregnancy is a rare but potentially life‐threatening complication. This case report and systematic review demonstrate that the condition can occur even in the early stages of gestation and may require intensive care management. Early administration of intravenous ampicillin and timely initiation of ventilatory support are critical to improving maternal and fetal outcomes. Given its low incidence but high severity, clinicians should maintain a high index of suspicion in pregnant patients presenting with systemic symptoms, particularly those with a history of consuming high‐risk foods, and reinforce early diagnostic and preventive strategies.

## Ethics Statement

As a case report involving educational and medical activities, it was exempt from requiring approval by the Institutional Review Board of the “Hospital Nacional Dos de Mayo.”

## Consent

The authors certify that they have obtained all appropriate patient consent forms. In the form, the patient has given consent for their images and other clinical information to be reported in the journal.

## Conflicts of Interest

The authors declare no conflicts of interest.

## Funding

No funding was received for this manuscript.

## Supporting information


**Supporting Information** Additional supporting information can be found online in the Supporting Information section. Table S1 provides the complete search strategies for each database, structured around three core concepts.

## Data Availability

The data that supports the findings of this study are available in the supporting information of this article.
