# Supplementary material for: Acute Respiratory Distress Syndrome Associated With Listeria monocytogenes in a Pregnant Woman: Case Report and Systematic Review
Source: Case Rep Crit Care. 2025 Oct 29;2025:9923135. doi: 10.1155/crcc/9923135 (PMC12569432; doi:10.1155/crcc/9923135)
Supplement: Supplementary file 1 — Supporting Information Additional supporting information can be found online in the Supporting Information section. Table S1 provides the complete search strategies for each database, structured around three core concepts. [file CRCC-2025-9923135-s001.docx]

## Supplementary Material

## Supplementary Table 1. Search strategy

| Database | Search strategy | Results |
| --- | --- | --- |
| Pubmed/ Medline | #1: "Listeriosis"[Mesh] OR Listeri*[TIAB]  #2: "Respiratory Distress Syndrome"[Mesh] OR “Respiratory Distress Syndrome*”[TIAB] OR “Shock Lung”[TIAB] OR ARDS[TIAB]  #3: "Pregnant Women"[Mesh] OR "Pregnancy"[Mesh] OR Pregnan*[TIAB] OR Gestation*[TIAB]  #4: #1 AND #2 AND #3 | 15 |
| Scopus | #1: ALL (Listeri*)  #2: TITLE-ABS-KEY (“Respiratory Distress Syndrome*” OR “Shock Lung” OR ARDS)  #3: TITLE-ABS-KEY (Pregnan* OR Gestation*)  #4: #1 AND #2 AND #3 | 63 |
| WOS | #1: ALL=(Listeri*)  #2: TS=(“Respiratory Distress Syndrome*” OR “Shock Lung” OR ARDS) OR AK=(“Respiratory Distress Syndrome*” OR “Shock Lung” OR ARDS) OR KP=(“Respiratory Distress Syndrome*” OR “Shock Lung” OR ARDS) OR TI=(“Respiratory Distress Syndrome*” OR “Shock Lung” OR ARDS) OR AB=(“Respiratory Distress Syndrome*” OR “Shock Lung” OR ARDS)  #3: TS=(Pregnan* OR Gestation*) OR AK=(Pregnan* OR Gestation*) OR KP=(Pregnan* OR Gestation*) OR TI=(Pregnan* OR Gestation*) OR AB=(Pregnan* OR Gestation*)  #4: #1 AND #2 AND #3 | 6 |
| Scielo | #1: ALL=(Listeri*)  #2: TS=(“Respiratory Distress Syndrome*” OR “Shock Lung” OR ARDS) OR AK=(“Respiratory Distress Syndrome*” OR “Shock Lung” OR ARDS) OR KP=(“Respiratory Distress Syndrome*” OR “Shock Lung” OR ARDS) OR TI=(“Respiratory Distress Syndrome*” OR “Shock Lung” OR ARDS) OR AB=(“Respiratory Distress Syndrome*” OR “Shock Lung” OR ARDS)  #3: TS=(Pregnan* OR Gestation*) OR AK=(Pregnan* OR Gestation*) OR KP=(Pregnan* OR Gestation*) OR TI=(Pregnan* OR Gestation*) OR AB=(Pregnan* OR Gestation*)  #4: #1 AND #2 AND #3 | 0 |
| Embase | #1: ('listeria'/exp OR listeria OR 'listeria' OR 'listeriosis'/exp OR 'l. monocytogenes infection' OR 'listeria infection' OR 'listeria infections' OR 'listeria monocytogenes infection' OR 'circling disease' OR 'infection by l. monocytogenes' OR 'infection by listeria monocytogenes' OR 'infection caused by l. monocytogenes' OR 'infection caused by listeria monocytogenes' OR 'infection due to l. monocytogenes' OR 'infection due to listeria monocytogenes' OR 'infection of listeria monocytogenes' OR 'listerellosis' OR 'listeriasis' OR 'listeriosis' OR 'lysteriosis' OR 'listeria monocytogenes'/exp OR 'corynebacterium infantisepticum' OR 'corynebacterium parvulum' OR 'erysipelothrix monocytogenes' OR 'listerella hepatolytica' OR 'listeria monocytogenes' OR 'bacterium monocytogenes' OR 'listeriosis monocytogenes')  #2: ('adult respiratory distress syndrome'/exp OR 'ards' OR 'acute respiration distress syndrome' OR 'acute respiratory disease syndrome' OR 'acute respiratory distress syndrome' OR 'adult respiration distress' OR 'adult respiratory distress' OR 'adult respiratory distress syndrome' OR 'lung shock' OR 'posttraumatic lung failure' OR 'posttraumatic pulmonary insufficiency' OR 'respiratory distress syndrome, acute' OR 'respiratory distress syndrome, adult' OR 'respiratory distress, adult' OR 'shock lung' OR 'respiratory distress syndrome'/exp OR 'rds' OR 'breathing distress syndrome' OR 'lung distress syndrome' OR 'pulmonary distress syndrome' OR 'respiration distress syndrome' OR 'respiratory distress syndrome')  #3: ('pregnancy'/exp OR 'child bearing' OR 'childbearing' OR 'gestation' OR 'gravidity' OR 'intrauterine pregnancy' OR 'labor presentation' OR 'labour presentation' OR 'pregnancy' OR 'pregnancy maintenance' OR 'pregnancy trimesters')  #4: #1 AND #2 AND #3 | 22 |
